# A52 GUT-INNERVATING TRPV1+ NEURONS DRIVE CHRONIC VISCERAL PAIN VIA MICROGLIAL P2Y12 RECEPTOR

**DOI:** 10.1093/jcag/gwab049.051

**Published:** 2022-02-21

**Authors:** M Defaye, N Abdullah, M Iftinca, A Hassan, F Agosti, Z Zhang, M Cumenal, G W Zamponi, C Altier

**Affiliations:** Physiology and Pharmacology, University of Calgary Cumming School of Medicine, Calgary, AB, Canada

## Abstract

**Background:**

Long-lasting changes in neural pain circuits precipitate the transition from acute to chronic pain in patients living with inflammatory bowel diseases (IBDs). While significant improvement in IBD therapy has been made to reduce inflammation, a large subset of patients continues to suffer throughout quiescent phases of the disease. Peripheral and central mechanisms contribute to the transition from acute to chronic pain during active disease and clinical remission. Lower mechanical threshold and hyperexcitability of visceral afferents induce gliosis in central pain circuits, leading to persistent visceral hypersensitivity (VHS). In the spinal cord, microglia, the immune sentinels of the central nervous system, undergo activation in multiple models of VHS. Using the Dextran Sodium Sulfate (DSS) model of colitis, we found that microglial G-CSF was able to sensitize colonic nociceptors that express the pain receptor TRPV1. While TRPV1+ nociceptors have been implicated in peripheral sensitization, their contribution to central sensitization via microglia remains unknown.

**Aims:**

Here we investigated the mechanisms of microglia activation to identify centrally acting analgesics for chronic IBD pain.

**Methods:**

Using Designer Receptors Exclusively Activated by Designer Drugs (DREADD) expressed in TRPV1-expressing visceral neurons that sense colonic inflammation, we tested whether neuronal activity was indispensable to control microglia activation and VHS. We then investigated the neuron-microglia signaling system involved in visceral pain chronification.

**Results:**

We found that chemogenetic inhibition of TRPV1+ visceral afferents prevents microglial activation in the spinal cord and subsequent VHS in colitis mice. In contrast, chemogenetic activation, in the absence of colitis, enhanced microglial activation associated with VHS. We identified a purinergic signaling mechanism mediated by neuronal ATP and microglial P2RY12 receptor, triggering VHS in colitis. Inhibition of P2RY12 prevented microglial reactivity and chronic VHS post-colitis.

**Conclusions:**

Overall, these data provide novel insights into the central mechanisms of chronic visceral pain and suggest that targeting microglial P2RY12 signaling could be harnessed to relieve pain in IBD patients who are in remission.

**Funding Agencies:**

CCC

